# Supplementary material for: High-Throughput Identification of Adapters in Single-Read Sequencing Data
Source: Biomolecules. 2020 Jun 8;10(6):878. doi: 10.3390/biom10060878 (PMC7356586; doi:10.3390/biom10060878)
Supplement: Supplementary file 1 [file biomolecules-10-00878-s001.zip › supplementary layout/Additional file 1.docx]

**Additional file 1, Figure S1:** Options in adapt_find tool.

**
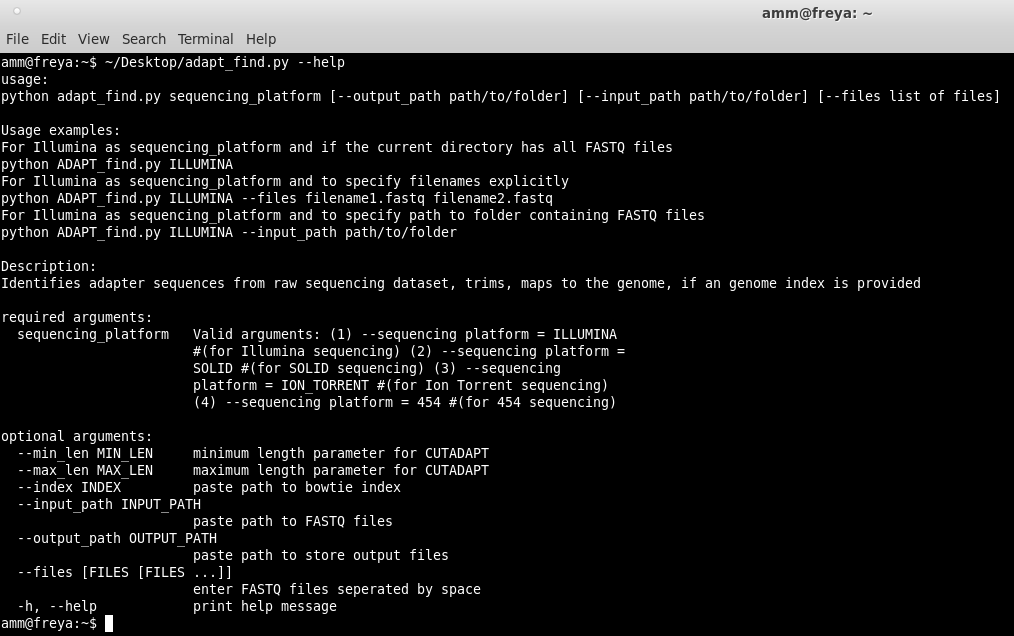
**

**Additional file 1, Figure S2**: Testing fastp on a publicly available dataset for identifying adapter sequence.


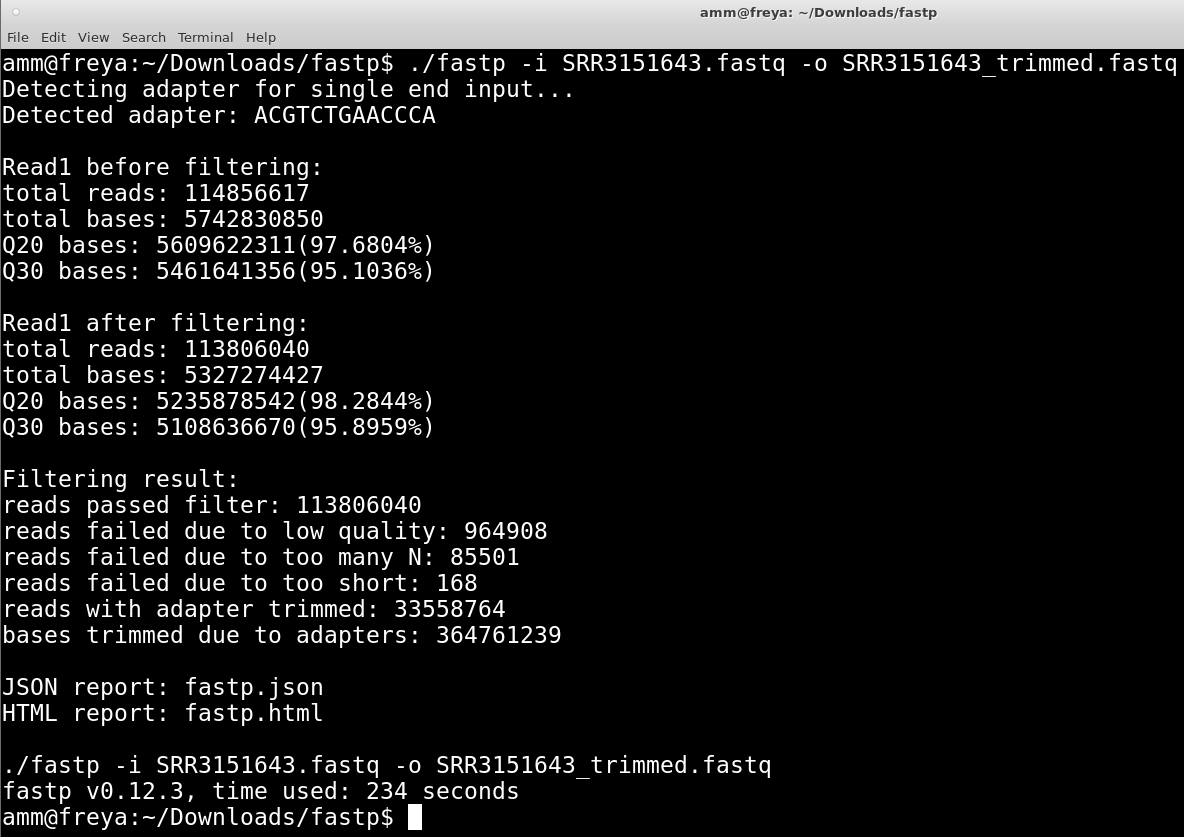


**Additional file 1, Figure S3**: Testing adapt_find on a publicly available dataset for identifying adapter sequence.


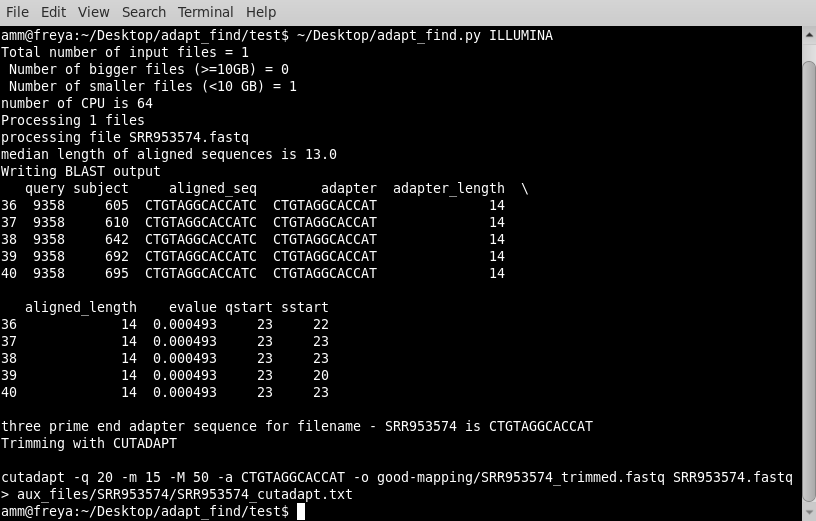


**Additional file 1, Figure S4**: Highlighted in green are the subject sequences of 5’ end adapters (ATCC) in in the dataset SRR578917. Nota bene: ATCC motifs are also found within biological sequences, in a distance from the 5’ end (here, in lines 4, 16, 28, 32 and 36).


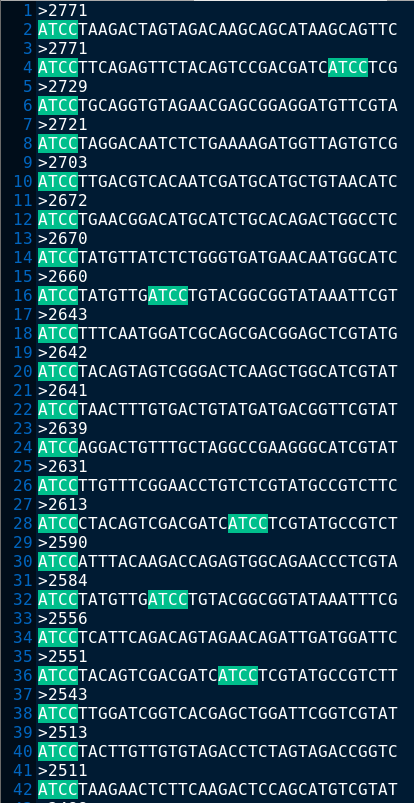


**Additional file 1, Figure S5**: Highlighted in green are the 3’ end adapters in the subject sequences (TGGAATTCTCGGGTGC) in the dataset SRR997335. Nota bene: sequences without identified adapter motif have either the adapter sequence modified or truncated.


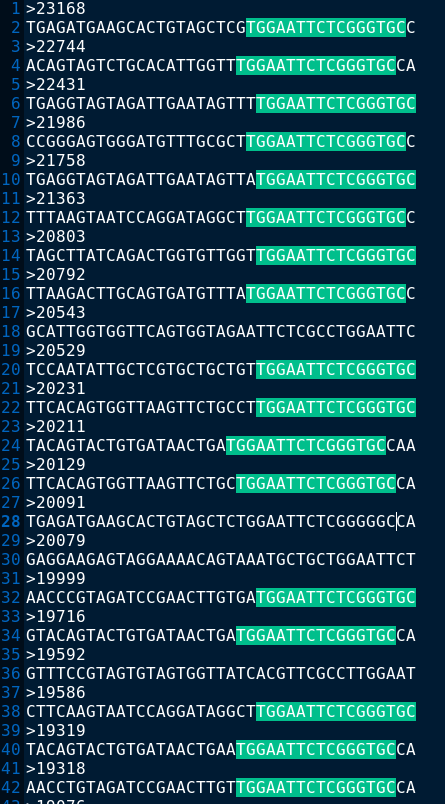


**Additional file 1, Figure S6**: Sample output from adapt_find for test datasets.


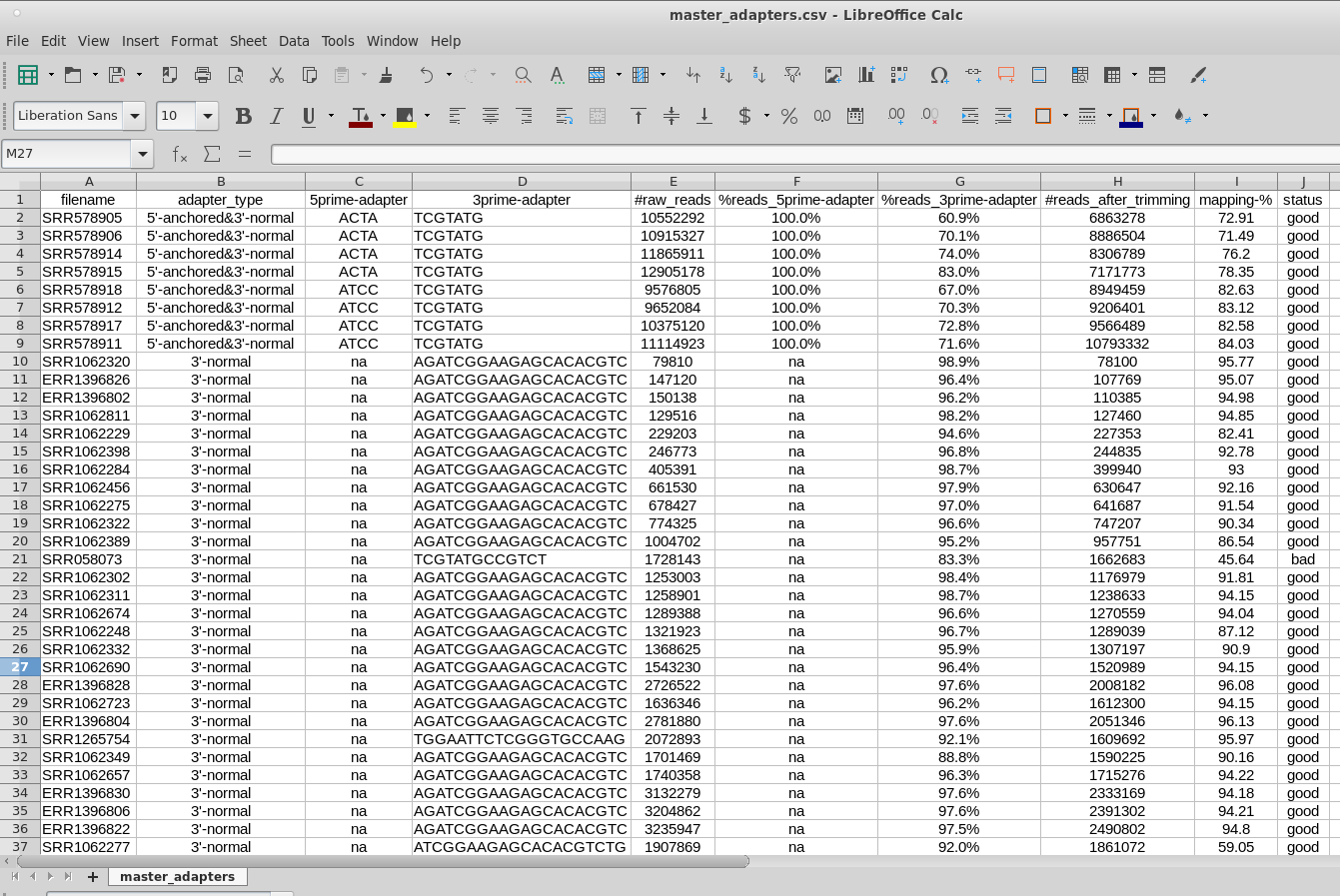


**Additional file 1, Table S1**: Optional arguments for adapt_find tool.

| **Argument** | **Default Option** |
| --- | --- |
| --min_len | 15 |
| --max_len | 50 |
| --index | None. If the path to the bowtie genome index files is specified, the percentage of number of reads mapping to genome after adapter trimming would be reported. |
| --input_path | Current working directory |
| --output_path | Current working directory |
| --files | None. If the files are located in different locations, the absolute path of the files can be specified. Note: if –input_path argument is specified, --files argument cannot be specified. If –files argument is specified, files in the current working directory will not be analyzed, even though the output directory will be the current working directory – unless a different output path is specified using --output_path argument. |
